# A novel neurotensin/xenin fusion peptide enhances β-cell function and exhibits antidiabetic efficacy in high-fat fed mice

**DOI:** 10.1042/BSR20211275

**Published:** 2021-08-26

**Authors:** Rachele A. Perry, Sarah. L. Craig, Victor A. Gault, Peter R. Flatt, Nigel Irwin

**Affiliations:** Ulster University, School of Pharmacy and Pharmaceutical Sciences, Diabetes Research Group, Coleraine, Northern Ireland, U.K.

**Keywords:** hybrid, incretin, Neurotensin, Xenin

## Abstract

Neurotensin and xenin possess antidiabetic potential, mediated in part through augmentation of incretin hormone, glucagon-like peptide-1 (GLP-1) and glucose-dependent insulinotropic polypeptide (GIP), action. In the present study, fragment peptides of neurotensin and xenin, acetyl-neurotensin and xenin-8-Gln, were fused together to create Ac-NT/XN-8-Gln. Following assessment of enzymatic stability, effects of Ac-NT/XN-8-Gln on *in vitro* β-cell function were studied. Subchronic antidiabetic efficacy of Ac-NT/XN-8-Gln alone, and in combination with the clinically approved GLP-1 receptor agonist exendin-4, was assessed in high-fat fed (HFF) mice. Ac-NT/XN-8-Gln was highly resistant to plasma enzyme degradation and induced dose-dependent insulin-releasing actions (*P*<0.05 to *P*<0.01) in BRIN-BD11 β-cells and isolated mouse islets. Ac-NT/XN-8-Gln augmented (*P*<0.001) the insulinotropic actions of GIP, while possessing independent β-cell proliferative (*P*<0.001) and anti-apoptotic (*P*<0.01) actions. Twice daily treatment of HFF mice with Ac-NT/XN-8-Gln for 32 days improved glycaemic control and circulating insulin, with benefits significantly enhanced by combined exendin-4 treatment. This was reflected by reduced body fat mass (*P*<0.001), improved circulating lipid profile (*P*<0.01) and reduced HbA1c concentrations (*P*<0.01) in the combined treatment group. Following an oral glucose challenge, glucose levels were markedly decreased (*P*<0.05) only in combination treatment group and superior to exendin-4 alone, with similar observations made in response to glucose plus GIP injection. The combined treatment group also presented with improved insulin sensitivity, decreased pancreatic insulin content as well as increased islet and β-cell areas. These data reveal that Ac-NT/XN-8-Gln is a biologically active neurotensin/xenin fusion peptide that displays prominent antidiabetic efficacy when administered together with exendin-4.

## Introduction

Neurotensin and xenin are regulatory peptides that share amino acid sequence homology, activate similar receptors and possess some notable parallels in their bioactivity profiles [1,2]. Both hormones stimulate appetite suppression [2–4] as well as modulate insulin secretion and glucose homeostasis [3,5]. Positive effects of neurotensin and xenin have been observed also on pancreatic β-cell growth and survival [4,6], together with prominent effects on metabolic regulation. In this regard, there is good evidence that neurotensin is co-expressed and co-released with the incretin hormone glucagon-like peptide-1 (GLP-1) from intestinal L-cells [4], whilst xenin is similarly secreted alongside glucose-dependent insulinotropic polypeptide (GIP) from enteroendocrine K-cells [2]. However, it should also be noted that neurotensin is also synthesised and secreted by enteroendocrine N-cells [7], suggesting that secretion of GLP-1 and NT is not fully paralleled *in vivo*. Nonetheless, neurotensin has recently been shown to augment the biological actions of GLP-1 [8], while xenin is well known to potentiate GIP-mediated bioactivity [9–11]. Thus, together neurotensin and xenin have the capacity to significantly enhance the biological actions of the incretin hormones. This is of particular interest in terms of the treatment of type 2 diabetes, given that a number of clinically approved antidiabetic therapeutics directly modulate the incretin system [12], with other agents believed to exert some of their benefits through this pathway [13,14].

Interestingly, the C-terminal portions of both neurotensin and xenin have been shown to retain similar bioactivity as their respective parent peptides [15,16]. This would suggest that shorter neurotensin and xenin peptides which are easier to synthesise and formulate, may have therapeutic promise for diabetes. One potential hindrance to the use of such truncated neurotensin or xenin peptides relates to their relatively brief biological half-lives [16,17]. However, enzymatic stability and subsequent prolongation of bioactivity, can be imparted through appropriate structural modification of these C-terminal truncated peptides [15,18]. As such, acetyl-neurotensin [8–13] and xenin-8-Gln represent fully characterised, enzymatic resistant, bioactive neurotensin and xenin peptide analogues [19,20]. To increase therapeutic applicability of gut hormone-derived peptides, generation of hybrid unimolecular peptides has been employed of late, through merging of the key bioactive amino acid sequences of the parent peptides [21–23]. Based on this knowledge, we have constructed a novel neurotensin/xenin hybrid peptide, namely Ac-NT/XN-8-Gln, by fusing together the amino acid sequences of acetyl-neurotensin [8–13] and xenin-8-Gln. The xenin component of the fusion peptide was positioned at the C-terminus, as this has been employed with good success for other xenin hybrid peptides [24–26].

In the current study, we initially examined plasma stability of Ac-NT/XN-8-Gln followed by assessment of *in vitro* insulinotropic actions alone, and in combination with GIP or GLP-1. In addition, the impact of Ac-NT/XN-8-Gln on β-cell proliferation and protection against apoptosis was also investigated in BRIN-BD11 β-cells. Following confirmation of intact bioactivity of Ac-NT/XN-8-Gln, beneficial metabolic effects of twice daily administration alone, or in combination with exendin-4, were evaluated in high-fat fed (HFF) mice.

## Materials and methods

### Peptides

All peptides (Table 1) were purchased from Syn Peptide (Shanghai, China) at greater than 95% purity. In-house confirmation of peptide purity and molecular weight was carried out by RP-HPLC and MALDI-TOF MS, as previously described [27].

**Table 1:** Amino acid sequence, MALDI-TOF MS analysis and enzymatic stability of xenin-8-Gln, acetyl-neurotensin(8-13) and Ac-NT/XN-8-Gln

Peptide	Sequence	Percentage intact peptide remaining (%)		
		2 h	4 h	8 h
Xenin-8-Gln	H-HIS-PRO-GLN-GLN-PRO-TRP-ILE-LEU-OH	100	98.3	94.1
Acetyl-neurotensin(8-13)	Ac-ARG-ARG-PRO-TYR-ILE-LEU-OH	98.2	94.4	89.6
Ac-NT/XN-8-Gln	Ac-ARG-ARG-PRO-TYR-ILE-LEU- HIS-PRO-GLN-GLN-PRO-TRP-ILE-LEU-OH	100	100	91.7

Peptide stability was assessed following 2-, 4- and 8-h incubation in murine plasma at 37°C. Degradation products were separated using HPLC, analysed by MS, and percentage degradation calculated from peak areas.

### Effects of peptides on *in vitro* insulin secretion

The *in vitro* insulin secretory activity of test peptides was examined in BRIN-BD11 cells, cultured and maintained as previously described [28]. For experimentation, BRIN-BD11 cells were seeded in 24-well plates at a cell density of 150000 cells/well, and allowed to attach overnight at 37°C. Following pre-incubation with Krebs–Ringer bicarbonate buffer (KRBB) (pH 7.4) supplemented with 0.5% (w/v) BSA and 1.1 mM glucose (40 min; 37°C), cells were then incubated with test peptides alone (10^−6^–10^−12^ M), or in combination with either GIP or GLP-1 (10^−7^ M), at 5.6 or 16.7 mM glucose, as appropriate for 20 min. Relatively high concentrations of GIP and GLP-1 were employed for *in vitro* studies and these hormones have been shown to be synthesised and secreted locally within islets [29], with our previous studies confirming that augmentation of the insulinotropic actions of 10^−7^ M GIP or GLP-1 is achievable [30,31]. Aliquots of assay buffer (200 μl) were collected and stored at −20°C prior to assessment of insulin concentrations by an in-house radioimmunoassay [32]. To confirm insulin secretory activity of Ac-NT/XN-8-Gln, pancreatic islets were isolated from lean C57BL/6 mice by collagenase digestion, as described previously [33] and insulin secretion determined as outlined above (16.7 mM glucose), but over a 60-min test incubation period.

### Effects of peptides on *in vitro* β-cell proliferation and protection against apoptosis

BRIN-BD11 β-cells were used to investigate effects of test peptides (10^−8^ and 10^−6^ M) on β-cell proliferation and protection against apoptosis. GLP-1 was employed as a positive control for all studies. Ki-67 immunostaining was used to assess effects on proliferation. Briefly, cells were seeded on to coverslips (40000 cells per coverslip) and cultured overnight (18 h; 37°C), in the presence of test peptides (10^−8^ and 10^−6^ M). Cells were then washed with PBS, and fixed using 4% paraformaldehyde. Following antigen retrieval with citrate buffer (90°C for 20 min), tissues were blocked using 1.1% BSA for 30 min. Cells were then incubated with Ki-67 primary antibody (1:500; Abcam, ab15580), followed by Alexa Fluor® 488 secondary antibody (1:400, Invitrogen, A-11008). Coverslips were washed with PBS, mounted on slides for viewing using a fluorescent microscope (Olympus System Microscope) and photographed by DP70 camera adapter system. Proliferation frequency was expressed as percentage of total cells analysed. For analysis of the ability of test peptides (10^−8^ and 10^−6^ M) to protect against apoptosis, cells were seeded as above, but apoptosis evoked through activation of caspase 3 and 7 activity by a luminogenic, tetrapeptide sequence DEVD Caspase-Glo® 3/7 substrate. Caspase induced cleavage of the substrate was then assessed by an ApoLive-Glow™ Multiplex assay kit, according to the protocol provided by the manufacturer (Promega, Wisconsin, U.S.A.).

### Animals

Studies were carried out using male NIH Swiss mice (12–14 weeks of age, Envigo Ltd, U.K.), housed individually in an air-conditioned room at 22 ± 2°C with a 12-h light:12-h dark cycle. Prior to experiments, animals were maintained on a high-fat diet (45% fat, 35% carbohydrate and 20% protein, Special Diet Services, U.K., with percent of total energy of 26.15 kJ/g) for 10 weeks. This diet resulted in progressive body weight gain and hyperglycaemia compared with control mice. Lean control animals were maintained on a standard rodent chow diet (10% fat, 30% protein and 60% carbohydrate, Trouw Nutrition, U.K., with percent of total energy of 12.99 kJ/g). All mice had *ad libitum* access to water and respective diets. Experiments were carried out in accordance with the U.K. Animal Scientific Procedures Act 1986 and approved by the University of Ulster Animal Welfare and Ethical Review Body (AWERB) under the UK Home Office project licence 2804. All animal studies were conducted in the Biomedical and Behavioural Research Unit (BBRU) at Ulster University, Coleraine.

### Subchronic studies

Twice daily i.p. injections of saline vehicle (0.9% w/v NaCl), exendin-4, Ac-NT/XN-8-Gln or a combination of both peptides (each peptide at 25 nmol/kg bw) were administered at 09:30 and 17:30 h for 32 days in HFF mice. Cumulative food intake, body weight, non-fasting glucose and insulin concentrations were monitored at regular intervals. HbA1c concentrations were measured on day 32. In addition, at the end of the treatment period, oral glucose tolerance (18 mmol/kg bw; p.o.), biological response to GIP (18 mmol/kg glucose in combination with native GIP (25 nmol/kg; i.p.) and insulin sensitivity (15 U/kg bw; i.p.) tests were performed. All test solutions were administered in a final volume of 5 ml/kg body weight.

### Terminal analyses

All animals were killed via CO_2_ gas inhalation and cervical dislocation. Percentage body fat mass was measured at study termination by DEXA scanning (PIXImus Densitometer; Inside Outside Sales, Fitchburg, WI, U.S.A.). Pancreatic insulin content was measured following hormone extraction (1.5% HCl, 75% ethanol and 23.5% H_2_O), as described previously [34]. For histological analyses, tissue was excised, immediately fixed using 4% paraformaldehyde and embedded in paraffin wax. Tissue sections were then deparaffinised, rehydrated and probed with primary antibodies, namely mouse anti-insulin antibody (1:500; Abcam, ab6995) and guinea-pig anti-glucagon antibody (PCA2/4, 1:400; raised in-house). Sections were incubated with secondary antibodies, Alexa Fluor 594 goat anti-mouse IgG (1:400) and Alexa Fluor 488 goat anti-guinea pig IgG (1:400). The slides were viewed under a FITC (488 nm) or TRITC filter (594 nm) using a fluorescent microscope (Olympus system microscope, model BX51) and photographed using a DP70 camera adapter system. Islet parameters were analysed using Cell^F^ image analysis software (Olympus Soft Imaging Solutions, GmbH).

### Biochemical analyses

Blood samples were collected from the tail vein of conscious mice into chilled fluoride/heparin glucose microcentrifuge tubes (Sarstedt, Numbrecht, Germany) at the time points indicated in the figures. Blood glucose was measured directly using a handheld Ascensia Contour blood glucose meter (Bayer Healthcare, Newbury, Berkshire, U.K.). HbA1c concentrations in whole blood were measured using A1cNow® kits (PTS diagnostics, Indiana, U.S.A.). Total and LDL-cholesterol, as well as blood triacylglycerol concentrations, were measured using a Hitachi Automated Analyser 912 (Boehringer Ingelheim, Mannheim, Germany). Plasma and pancreatic insulin were assayed by a modified dextran-coated charcoal radioimmunoassay, as described previously [32].

### Statistical analysis

Statistical analysis was performed using GraphPad Prism (Version 5). Results are expressed as means ± SEM and data compared using repeated-measures ANOVA followed by the Student–Newman–Keuls *post hoc* test. Unpaired Student’s *t* test was used where appropriate. Area under curve (AUC) and area above curve (AAC) analyses were calculated using trapezoidal rule with baseline subtraction. Groups of data were significantly different if *P*<0.05.

## Results

### Plasma enzyme stability

When incubated together with murine plasma, Ac-NT/XN-8-Gln remained 100% intact up to and including 4 h, and over 90% peptide intact over an extended 8-h incubation period (Table 1). Native GLP-1 was employed as a positive control in this system, and was fully degraded by 4 h (data not shown).

### Effects of Ac-NT/XN-8-Gln on insulin release from BRIN-BD11 cells

**Figure 1:**
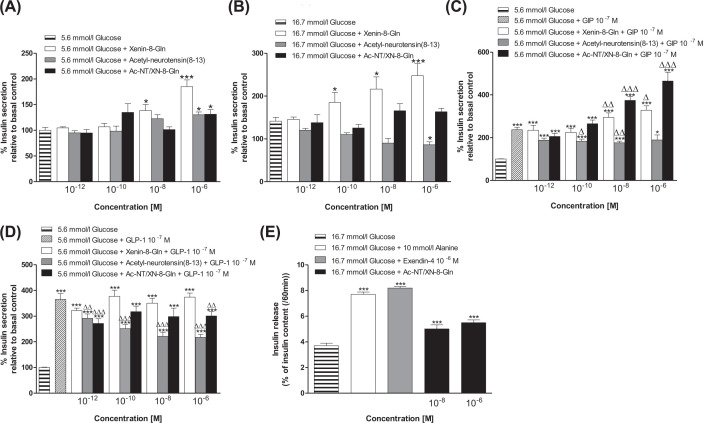
Acute effects of acetyl-neurotensin(8-13), xenin-8-Gln, Ac-NT/XN-8-Gln alone and in combination with GIP or GLP-1, on insulin release from BRIN-BD11 cells and isolated mouse islets BRIN-BD11 cells were incubated (20 min) with a range of concentrations (10^−12^ to 10^−6^ M) of acetyl-neurotensin(8-13), xenin-8-Gln or Ac-NT/XN-8-Gln alone (**A**,**B**) or in combination with GIP (**C**) or GLP-1 (**D**) in the presence of (A,C,D) 5.6 or (B) 16.7 mM glucose. (**E**) Murine islets were incubated (60 min) with Ac-NT/XN-8-Gln (10^−8^ and 10^−6^ M) in the presence 16.7 mM glucose. (A–E) Insulin release was measured using radioimmunoassay. Values represent means ± SEM (*n*=8 for (A–D) and *n*=5 for (E)). *P<0.05 and ***P<0.001 compared with respective glucose controls. ^∆^*P*<0.05, ^∆∆^*P*<0.01 and ^∆∆∆^*P*<0.001 compared with respective 10^−7^ M GIP or GLP-1 control.

The insulinotropic actions of Ac-NT/XN-8-Gln, and respective parent peptides, at 5.6 and 16.7 mM glucose were investigated in BRIN-BD11 cells. At 5.6 mM glucose, xenin-8-Gln increased (*P*<0.05 to *P*<0.001) insulin secretion at concentrations of 10^−8^ M and above, whilst acetyl-neurotensin [8–13] and Ac-NT/XN-8-Gln evoked a significant (*P*<0.05) increase in insulin secretion at 10^−6^ M (Figure 1A). Interestingly, at 16.7 mM glucose only xenin-8-Gln retained significant insulinotropic actions, whereas acetyl-neurotensin [8–13] inhibited (*P*<0.05) glucose-stimulated insulin secretion (Figure 1B). Xenin-8-Gln augmented (*P*<0.05 to *P*<0.01) GIP-induced insulin secretion but had no impact on GLP-1 evoked insulin release, with Ac-NT/XN-8-Gln possessing a similar, but more prominent, bioactivity profile in the presence of GIP (Figure 1C,D). Interestingly, acetyl-neurotensin [8–13] had no effect on GIP-induced insulin release and appeared to inhibit (*P*<0.05) GLP-1 related insulin secretion (Figure 1C,D). Prominent (*P*<0.01) insulinotropic actions of Ac-NT/XN-8-Gln were then confirmed in isolated mouse islets at 16.7 mM glucose (Figure 1E).

### Effects of Ac-NT/XN-8-Gln on β-cell proliferation and protection against apoptosis

**Figure 2:**
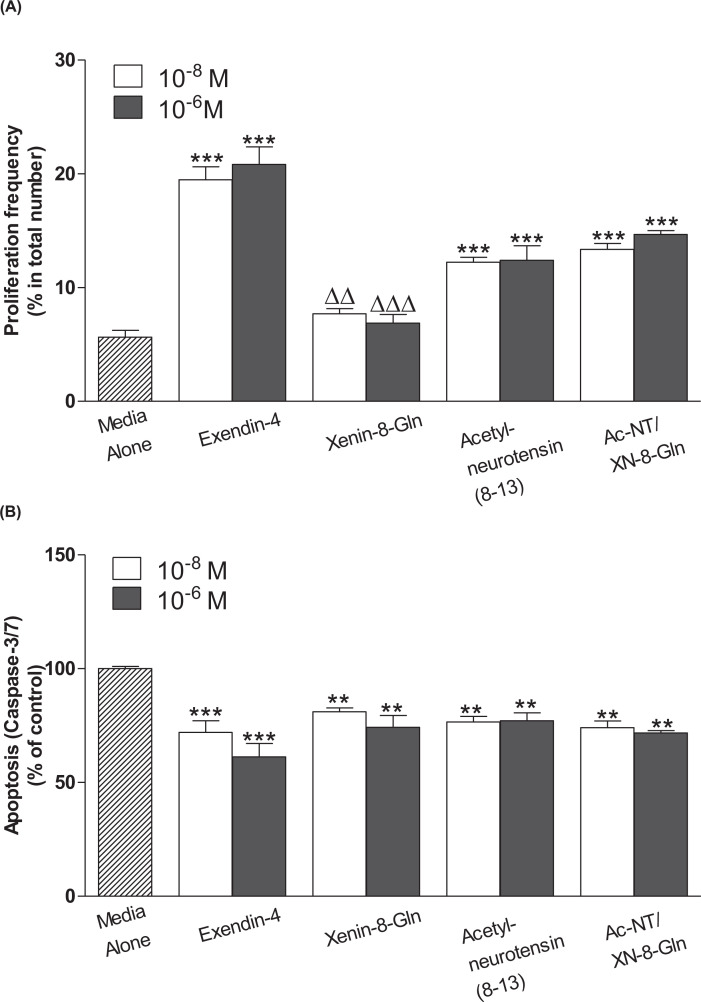
Effect of acetyl-neurotensin(8-13), xenin-8-Gln and Ac-NT/XN-8-Gln on β-cell proliferation and protection against apoptosis BRIN-BD11 cells were incubated overnight (18 h) with exendin-4, acetyl-neurotensin(8-13), xenin-8-Gln or Ac-NT/XN-8-Gln (each at 10^−8^ and 10^−6^ M). (**A**) Proliferation was measured using Ki-67 immunocytochemistry. (**B**) Caspase-3/7 activation was detected by luminescence. (**A**) Values represent means ± SEM (*n*=3–4). **P<0.01 and ***P<0.001 compared with respective media control. ^∆∆^*P*<0.01 and ^∆∆∆^*P*<0.001 compared with respective Ac-NT/XN-8-Gln. (**B**) Values are mean ± SEM (*n*=3). **P<0.01 and ***P<0.001 compared with untreated control culture.

Similar to exendin-4, but with some reduced efficacy, acetyl-neurotensin [8–13] and Ac-NT/XN-8-Gln significantly (*P*<0.001) augmented BRIN-BD11 β-cell proliferation at concentrations of 10^−8^ and 10^−6^ M (Figure 2A). Interestingly, in the current setting xenin-8-Gln was devoid of benefits on β-cell proliferation, with the actions of Ac-NT/XN-8-Gln being significantly (*P*<0.05 to *P*<0.01) superior to xenin-8-Gln (Figure 2A). In relation to protection against apoptosis, all test peptides protected against (*P*<0.01 to *P*<0.001) BRIN-BD11 β-cell apoptosis at 10^−8^ and 10^−6^ M when compared with control cultures (Figure 2B).

### Effects of twice-daily administration of Ac-NT/XN-8-Gln and exendin-4 alone, or in combination, on body weight, food intake, glucose, insulin and HbA1c concentrations in HFF mice

**Figure 3:**
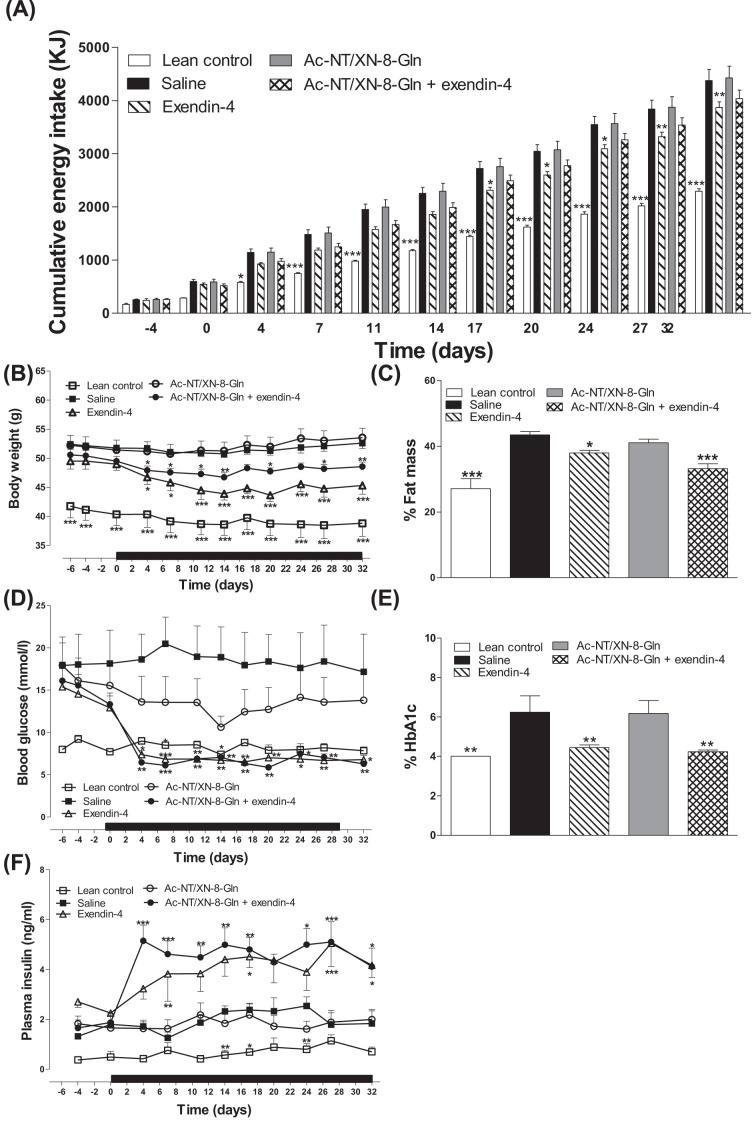
Effects of twice-daily administration of Ac-NT/XN-8-Gln and exendin-4 alone, or in combination, on body weight, food intake, glucose, insulin and HbA1c concentrations in HFF mice (**A**,**B**,**D**,**F**) Parameters were measured for 6 days before and 32 days during (indicated by black horizontal line in panels B,D,F) twice-daily treatment with saline, exendin-4, Ac-NT/XN-8-Gln or a combination of both (each at 25 nmol/kg) in HFF mice. (**C**,**E**) Parameters were assessed on day 32. Values represent mean ± SEM (*n*=6–8). *P<0.05, **P<0.01 and ***P<0.001 compared with HFF saline control mice.

Twice-daily administration of exendin-4 alone to HFF mice reduced (*P*<0.05 to *P*<0.01) cumulative energy intake from day 17 onwards when compared with HFF control mice, an effect that was not observed with any of the other treatment modalities (Figure 3A). Body weight was significantly decreased (*P*<0.05 to *P*<0.001) in mice receiving exendin-4 alone, or in combination with Ac-NT/XN-8-Gln (Figure 3B), but there was no obvious benefit of adding Ac-NT/XN-8-Gln to exendin-4 therapy in terms of overall body weight change (Figure 3B). Moreover, body fat mass was especially reduced (*P*<0.001) in HFF mice treated with Ac-NT/XN-8-Gln in combination with exendin-4 (Figure 3C). Non-fasting blood glucose levels were declined to levels similar to that of lean controls in HFF mice treated with exendin-4 alone, or in combination with Ac-NT/XN-8-Gln on day 4, and remained at normoglycaemic levels throughout in these groups of HFF mice (Figure 3D). In full agreement, HbA1c concentrations on day 32 were identical with lean controls in HFF mice treated with exendin-4 alone, or in combination with Ac-NT/XN-8-Gln (Figure 3E). Similarly, circulating insulin was elevated (*P*<0.05 to *P*<0.001) only in HFF mice receiving exendin-4 alone, or in combination with Ac-NT/XN-8-Gln (Figure 3F).

### Effects of twice-daily administration of Ac-NT/XN-8-Gln and exendin-4 alone, or in combination, on glucose tolerance, metabolic response to GIP, insulin sensitivity and lipid status in HFF mice

**Figure 4:**
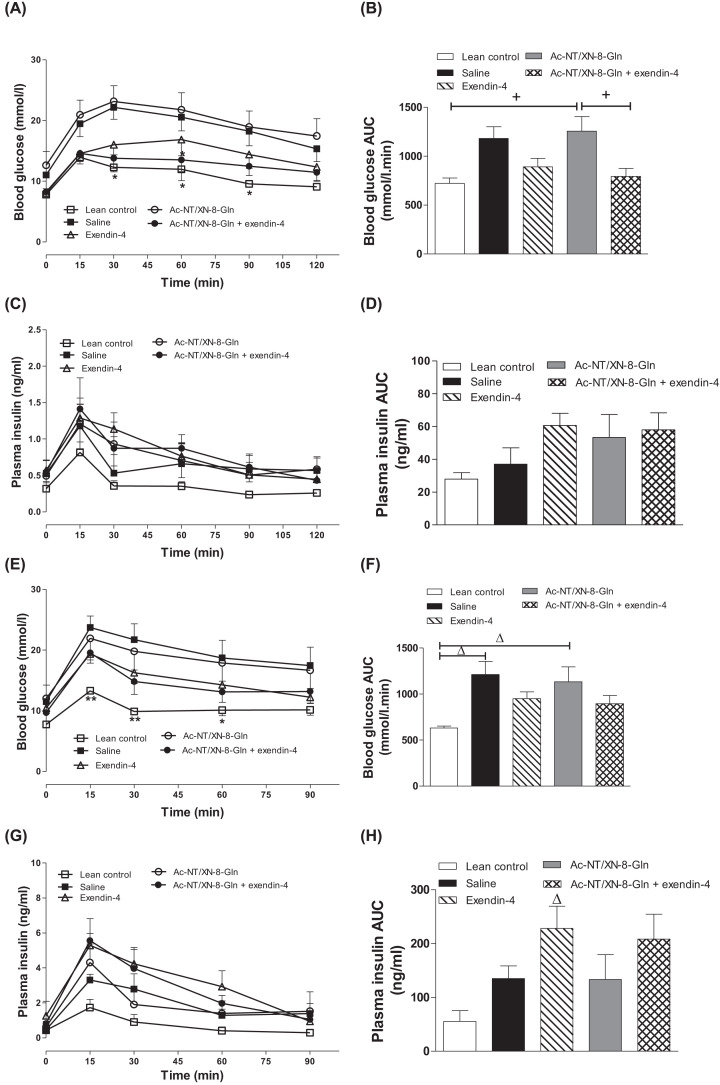
Effects of twice-daily administration of Ac-NT/XN-8-Gln and exendin-4 alone, or in combination, on glucose tolerance and metabolic response to GIP Tests were performed following 32 days twice-daily i.p. administration of saline, exendin-4, Ac-NT/XN-8-Gln or a combination of both peptides (each at 25 nmol/kg bw) in 10 h fasted HFF mice. (**A**–**D**) Blood glucose (A) and plasma insulin (C) were measured prior to and after oral administration of glucose alone (18 mmol/kg bw). (**E**–**H**) Blood glucose (E) and plasma insulin (G) were measured prior to and after i.p. administration of glucose (18 mmol/kg bw) in combination with GIP (25 nmol/kg bw). (B,D,F,H) Corresponding 0–120 min AUC values are also shown. Values represent mean ± SEM (*n*=6–8). *P<0.05 and **P<0.01 compared with HFF saline control mice. ^+^*P*<0.05 compared with Ac-NT/XN-8-Gln treated HFF mice. ^∆^*P*<0.05 compared with lean saline control mice. Where appropriate to aid interpretation, lines are used to indicate significance between groups on the bar graphs.

**Figure 5:**
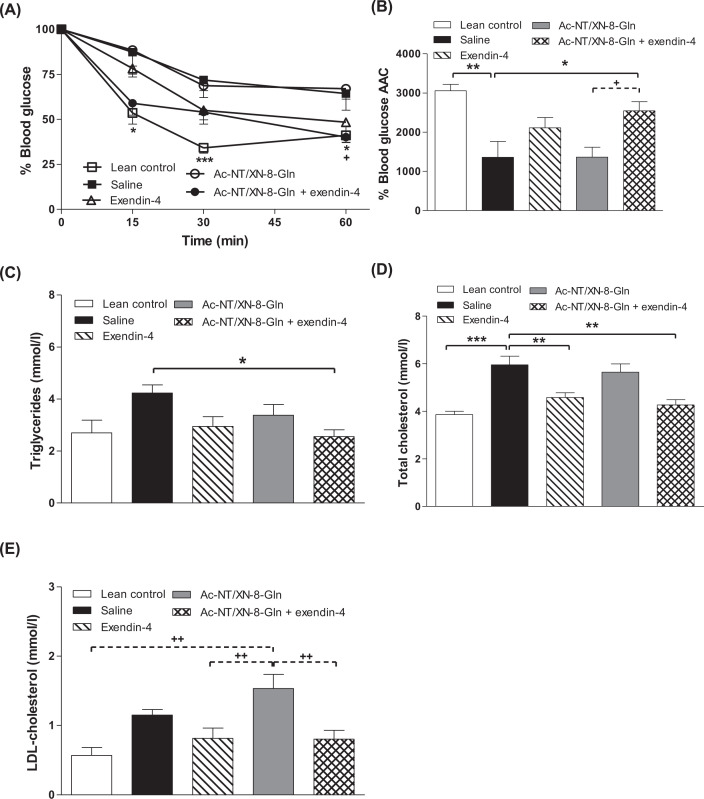
Effects of twice-daily administration of Ac-NT/XN-8-Gln and exendin-4 alone, or in combination, on insulin sensitivity and lipid status in HFF mice Tests were performed following 32 days twice-daily i.p. administration of saline, exendin-4, Ac-NT/XN-8-Gln or a combination of both peptides (each at 25 nmol/kg bw) in HFF mice. (**A**,**B**) Blood glucose was measured prior to and after i.p. administration of insulin (25 U/kg bw), with 0–60 min AAC values also shown. (**C**–**E**) Effects of test peptides on circulating (C) triglycerides, (D) total- and (E) LDL-cholesterol concentrations. Values are mean ± SEM (*n*=6–8). *P<0.05 **P<0.01 and ***P<0.001 compared with HFF saline control mice. ^+^*P*<0.05 and ^++^*P*<0.01 compared with Ac-NT/XN-8-Gln treated HFF mice. Where appropriate to aid interpretation, lines are used to indicate significance between groups on the bar graphs.

Following an oral glucose challenge, individual and 0–120-min AUC glucose levels were significantly decreased (*P*<0.05) only in mice treated with Ac-NT/XN-8-Gln in combination with exendin-4 when compared with HFF saline treated controls, with values similar to lean control mice (Figure 4A,B). In this respect, combination treatment was more effective than either parent peptide alone. There was a tendency for glucose-induced insulin secretion to be augmented in all HFF treatment groups, but this failed to reach significance (Figure 4C,D). Strikingly similar observations were made in response to glucose plus GIP injection, with only the combination treatment group evoking significant (*P*<0.05) metabolic benefits of GIP (Figure 4E–H), and superior to Ac-NT/XN-8-Gln or exendin-4 alone. These improvements in metabolism were associated with significantly improved (*P*<0.05) insulin sensitivity in the combined treatment group, that was not apparent with either individual peptide treatment (Figure 5A,B). In keeping with benefits of combined therapy, treatment of Ac-NT/XN-8-Gln in combination with exendin-4 reduced (*P*<0.05) circulating triacylglycerols (Figure 5C). In addition, both exendin-4 alone, and in combination with Ac-NT/XN-8-Gln, decreased (*P*<0.01) total cholesterol concentrations (Figure 5D), with LDL-cholesterol also decreased (*P*<0.01) in these groups of mice when compared with treatment with Ac-NT/XN-8-Gln alone (Figure 5E).

### Effects of twice-daily administration of Ac-NT/XN-8-Gln and exendin-4 alone, or in combination, on pancreatic insulin content and islet histology in HFF mice

**Figure 6:**
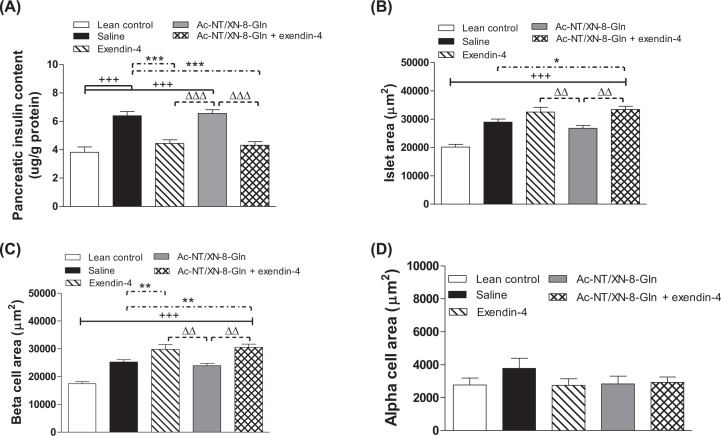
Effects of twice-daily administration of Ac-NT/XN-8-Gln and exendin-4 alone, or in combination, on pancreatic insulin content and islet histology in HFF mice Effects of 32 days twice-daily i.p. administration of saline, exendin-4, Ac-NT/XN-8-Gln or a combination of both peptides (each at 25 nmol/kg bw) on (**A**) pancreatic insulin content as well as (**B**) islet, (**C**) β-cell and (**D**) α-cell areas in HFF mice. Values represent mean ± SEM (*n*=6–8). ^+++^*P*<0.001 compared with lean control. *P<0.05, **P<0.01 and ***P<0.001 compared with HFF saline control mice. ^ΔΔ^*P*<0.01 and ^ΔΔΔ^*P*<0.001 compared with Ac-NT/XN-8-Gln treated HFF mice. Where appropriate to aid interpretation, lines are used to indicate significance between groups on the bar graphs.

Treatment with exendin-4 alone and in combination with Ac-NT/XN-8-Gln for 32 days decreased (*P*<0.001) pancreatic insulin content when compared with HFF control mice, with levels similar to that of lean controls (Figure 6A). HFF mice had significantly (*P*<0.001) increased islet area compared with lean controls (Figure 6B), that was associated with characteristic adaptive increases (*P*<0.001) in β-cell area (Figure 6C). Treatment with exendin-4 alone, and even more so in combination with Ac-NT/XN-8-Gln, further increased (*P*<0.05 to *P*<0.01) islet and β-cell areas (Figure 6B,C). Interestingly, although there was a tendency for α-cell area to be decreased by each treatment regimen, there were no significant differences in α-cell area between all groups of mice (Figure 6D).

## Discussion

Original early work demonstrated direct additive benefits of combined activation of GIP and xenin signalling pathways on pancreatic β-cell function and metabolism [10], which have been largely confirmed by others [24,35]. More recently, additive, or potentially synergistic, positive effects of combined GLP-1 and neurotensin signalling have also been evidenced [8]. Indeed, there is also suggestion that xenin can directly affect GLP-1 secretion and action [25,36], with neurotensin having a potentially comparable effect on GIP activity [37]. Taken together, neurotensin and xenin have the combined ability to modulate and augment the incretin effect, with obvious therapeutic implications for diabetes. This is even more apparent, given that highly prominent preclinical benefits of incretin enhancer drugs have not been fully translated to the clinic. Thus, the present study was designed to incorporate the metabolic advantages of neurotensin and xenin within a single unimolecular peptide, namely Ac-NT/XN-8-Gln, with subsequent characterisation of biological activity and assessment of ability to augment the antidiabetic efficacy of an established incretin mimetic drug.

As expected, Ac-NT/XN-8-Gln was highly resistant to enzymatic breakdown, in keeping with enhanced stability of the parent peptides acetyl-neurotensin [8–13] and xenin-8-Gln [19,20]. We were unable to perform *in vivo* pharmacokinetic analysis of Ac-NT/XN-8 as the specific assay required for such experiments is currently unavailable. Encouragingly however, Ac-NT/XN-8-Gln displayed prominent *in vitro* and *ex vivo* insulin secretory actions, and unlike neurotensin-related peptides [38], insulinotropic actions were evident under hyperglycaemic conditions. Thus, xenin peptides have been consistently shown to promote insulin secretion at both basal and elevated glucose levels [39,40], whereas neurotensin possesses reduced insulin secretory effectiveness at increased glucose concentrations [41]. Moreover, Ac-NT/XN-8-Gln imparted independent benefits on β-cell growth and survival as has been demonstrated previously with both native neurotensin and xenin [6,42]. Positive effects on pancreatic β-cell function and turnover are consistent with local synthesis and secretion of both hormones within the endocrine pancreas, and related essential physiological actions [2,4]. Thus, immunohistochemical analyses confirm the presence of both neurotensin and xenin immunoreactivity locally within islets [6]. Interestingly, in our experimental system, we were unable to recapitulate the potentiating effect of neurotensin on GLP-1 bioactivity [4,7], with acetyl-neurotensin [8–13] and Ac-NT/XN-8-Gln both appearing to perturb GLP-1 induced insulin secretion. However, in accord with previous observations [20], xenin-8-Gln clearly augmented GIP stimulated insulin secretion, with Ac-NT/XN-8-Gln fully replicating this beneficial effect, thus Ac-NT/XN-8-Gln may function more like xenin at the level of the pancreatic β-cell. In this regard, the receptor activation profile of Ac-NT/XN-8-Gln requires further detailed investigation, especially since the exact receptor that xenin signals through has not been fully established to date [35]. Thus, use of commercially available neurotensin receptor antagonists, or receptor knockout cell lines, may be useful to help uncover the receptors that are positively modulated by Ac-NT/XN-8-Gln. However, at the level of the pancreatic β-cell, it is clear that Ac-NT/XN-8-Gln retains ability to activate similar cell signaling pathways as the parent peptides, and we assume that this is also true in other tissues that express neurotensin or xenin receptors [2].

Following confirmation of enzymatic stability and preserved bioactivity of Ac-NT/XN-8-Gln, a chronic 32-day investigation of Ac-NT/XN-8-Gln antidiabetic actions alone, and in combination with the clinically approved GLP-1 mimetic, exendin-4, was conducted in the HFF mice. An additional group receiving exendin-4 alone was included as control. Although neurotensin and xenin are known to exert appetite suppressive actions [4,20,43], similar to GLP-1 [44], only treatment with exendin-4 alone decreased energy intake in HFF mice in the current setting. This was associated with clear reductions in body weight, which were also apparent, although to a lesser extent, with combined Ac-NT/XN-8-Gln injection. Indeed, exendin-4 induced benefits on circulating glucose levels were not improved by supplementation therapy with Ac-NT/XN-8-Gln. More interestingly however, the most prominent reduction in body fat content was noted in the combined treatment group, suggesting important benefits on lipid metabolism and body composition by co-activation of GLP-1, neurotensin and xenin signalling pathways in HFF mice. In agreement, whilst circulating lipid profile of HFF mice was improved by all treatment regimens, only combined therapy reduced triacylglycerol concentrations. In this regard, originally it was believed that xenin acts on adipose tissue to stimulate lipolysis [45], but more recent studies reveal direct lipogenic and adipocyte differentiation actions of xenin [46]. In agreement, an elegant study has suggested that neurotensin can also promote lipid accumulation [47]. However, activation of all three hormone signalling pathways appears to lead to reduced lipid deposition. In some agreement, interaction between xenin and GIP pathways within adipocytes has previously been noted to induce responses that contrast with effects of either hormone alone [46].

Decreased adiposity would be expected to improve insulin sensitivity [48], and this was very much apparent in HFF mice treated with a combination of Ac-NT/XN-8-Gln and exendin-4. Whilst GLP-1 is well recognised to improve insulin action [49], similar observations have also been made with xenin-based peptides [2]. Moreover, neurotensin is believed to be an important driving factor behind the improvement of insulin sensitivity in obese patients after metabolic surgery [50]. The same combined treatment group also displayed significantly improved glucose handling and glucose-lowering actions of GIP when compared with all other groups of HFF mice, despite no obvious augmentation of corresponding glucose- or GIP-induced insulin secretion, again suggesting improvement of insulin action in peripheral tissues. Additive benefits of Ac-NT/XN-8-Gln and exendin-4 to improve glucose disposal could also be linked with insulin-independent glucose-lowering actions of each hormone [20,49,51], but this would require further detailed investigation. However, pancreatic insulin stores were decreased in HFF mice treated with Ac-NT/XN-8-Gln in combination with exendin-4, implying reduced metabolic demand. Despite this, the characteristic expansion of pancreatic β-cells by sustained high-fat feeding in mice [52] was amplified by combined therapy, and likely linked to the increase of circulating insulin witnessed in these mice, although it should be noted that circulating insulin levels were also similarly increased in mice receiving exendin-4 alone. Such observations are in harmony with GLP-1 mediated benefits on β-cell mass [53], as well as in direct agreement with our *in vitro* observations with Ac-NT/XN-8-Gln.

In conclusion, the present study has demonstrated that the novel hybrid peptide Ac-NT/XN-8-Gln is biologically active and can substantially augment exendin-4 benefits in HFF mice, especially with regard to glucose handling, insulin action and lipid metabolism. Although further studies are required to uncover the underlying molecular mechanisms, it is evident that co-activation of GLP-1, neurotensin and xenin pathways represents an antidiabetic treatment option that merits further consideration.

## Data Availability

The authors declare that the data supporting the findings of the present study are available within the article. Any additional raw data supporting the conclusions of this article will be made available by the authors, without undue reservation.

## Abbreviations

AUC: area under curve
GIP: glucose-dependent insulinotropic polypeptide
GLP-1: glucagon-like peptide-1
HFF: high-fat fed
